# The impact of opioid administration on the incidence of postanaesthetic colic in horses

**DOI:** 10.3389/fpain.2024.1347548

**Published:** 2024-02-19

**Authors:** Rhea Haralambus, Michaela Juri, Anna Mokry, Florien Jenner

**Affiliations:** Equine Surgery Unit, University Equine Hospital, Department of Companion Animals and Horses, University of Veterinary Medicine Vienna, Vienna, Austria

**Keywords:** opioids, morphine, butorphanol, methadone, horse, equine, colic

## Abstract

Effective management of postoperative pain is essential to ensure patient welfare, reduce morbidity and optimize recovery. Opioids are effective in managing moderate to severe pain in horses but concerns over their adverse effects on gastrointestinal (GI) motility and associated increased colic risk limit their widespread use. Studies investigating the impact of systemic opioids on both GI motility and colic incidence in horses have yielded inconclusive outcomes. Therefore, this retrospective study aims to assess the influence of systemic administration of butorphanol, morphine, and methadone on post-anaesthetic colic (PAC) incidence. Horses undergoing general anaesthesia for non-gastrointestinal procedures that were hospitalized for at least 72 h post-anaesthesia were included in this study. Anaesthetised horses were stratified by procedure type into horses undergoing diagnostic imaging without surgical intervention, emergency or elective surgery. In addition, patients were grouped by opioid treatment regime into horses receiving no opioids, intraanaesthetic, short- (<24 h) or long-term (>24 h) postoperative opioids. Administered opioids encompassed butorphanol, morphine and methadone. The number of horses showing signs of colic in the 72 h after anaesthesia was assessed for each group. A total of 782 horses were included, comprising 659 undergoing surgical procedures and 123 undergoing diagnostic imaging. The overall PAC incidence was 15.1%. Notably, horses undergoing diagnostic imaging without surgery had a significantly lower PAC rate of 6.5% compared to those undergoing surgery (16.7%, *p* = 0.0146). Emergency surgeries had a significantly lower PAC rate of 5.8% compared to elective procedures (18%, *p* = 0.0113). Of the 782 horses, 740 received intraoperative opioids and 204 postoperative opioids, 102 of which long-term (≥24 h). Neither intraoperative (*p* = 0.4243) nor short-term postoperative opioids (*p* = 0.5744) increased PAC rates. Notably, only the long-term (≥24 h) administration of morphine significantly increased PAC incidence to 34% (*p* = 0.0038). In contrast, long-term butorphanol (5.3% PAC, *p* = 0.8482) and methadone (18.4% PAC, *p* = 0.6161) did not affect PAC rates. In summary, extended morphine administration was the only opioid treatment associated with a significantly increased risk of PAC.

## 1 Introduction

Postoperative pain is prevalent among the majority of patients undergoing surgical procedures. Effective pain management is crucial to mitigate suffering, reduce morbidity, and facilitate recovery and rehabilitation. Although the analgesic properties of opioids and opiates, such as butorphanol, buprenorphine, methadone, and morphine, are well-established, their use in managing perioperative and post-traumatic pain in equine patients is limited by concerns about potential adverse gastrointestinal side effects (1–9). Constipation is a widely recognized side effect of opioid treatment in all species, affecting up to 95% of human patients, attributed to diminished coordinated motility, prolonged transit time, enhanced fluid absorption from intestinal contents and decreased secretion of fluids and electrolytes into the intestinal lumen (10–20). While opioids provide analgesia by stimulating *μ*-, *κ*- and *δ*-opioid receptors in the brain, the dorsal horn of the spinal cord and peripheral tissues, the activation of opioid receptors within the gastrointestinal tract can decrease motility and induce alterations in the secretion, absorption and transport of electrolytes and fluids (9, 11–17, 21–24).

Post-anaesthetic colic (PAC) represents a common complication in equine patients, with reported incidence rates reaching up to 21.1% (5, 6, 25–28). Research into risk factors for the development of PAC and the influence of systemic opioids on gastrointestinal (GI) motility has produced equivocal results, ranging from decreased risk (25), no elevated risk (6, 26, 27, 29–32) to a fourfold rise in colic cases following perioperative opioid administration (5). The inconsistency in these outcomes could be attributed to differences in the GI side effects associated with various opioid agonists and variations in the dosage, frequency, administration method, and duration of opioid use across these studies.

Butorphanol, morphine, methadone and hydromorphone are commonly used in equine analgesia and their pharmacokinetics and pharmacodynamics have been thoroughly investigated (2, 24, 29, 30, 32, 33). Butorphanol, a synthetic strong *κ*-opioid receptor agonist and weak *μ*-opioid receptor antagonist (2, 34–38), has been observed to induce a transient reduction in gastrointestinal motility in anesthetized horses when used as CRI at a dosage of 13 μg/kg/h (34). In contrast, pre- or intraoperative butorphanol administration at a mean dosage of 0.007 mg/kg and 0.05 mg/kg respectively has been reported to reduce the risk of PAC (25).

Similarly, morphine, a *μ*-opioid agonist, has been shown to decrease gastrointestinal propulsive motility and to increase PAC rates after orthopaedic surgery four-fold when administered intravenously at a dosage of 0.08–0.3 mg/kg compared to no opioid or butorphanol (5, 9, 35, 39, 40). Conversely, two other studies found no association between peri-anaesthetic intravenous morphine administration at a dosage of 0.1–0.17 mg/kg and increased PAC risk (6, 41). Notably, the administration of epidural morphine after laparoscopy has been demonstrated to provide effective pain relief without compromising gastrointestinal motility (42).

Methadone, a synthetic *μ*-and *δ*-agonist with *N*-methyl-d-aspartate (NMDA) antagonist activity and the ability to inhibit serotonin and noradrenaline uptake, is not extensively approved for equine use and consequently, its utilization is less prevalent compared to morphine or butorphanol. Although it has also been shown to reduce borborygmi (2), the potential influence of perianaesthetic methadone administration on the occurrence of post-anaesthetic colic (PAC) remains unexplored.

Therefore, this study aims to assess and compare the impact of intra- and postoperative administration of the commonly used opioids—butorphanol, morphine and methadone—on the incidence of PAC in equine patients.

## 2 Materials and methods

### 2.1 Horses

**Figure 1:**
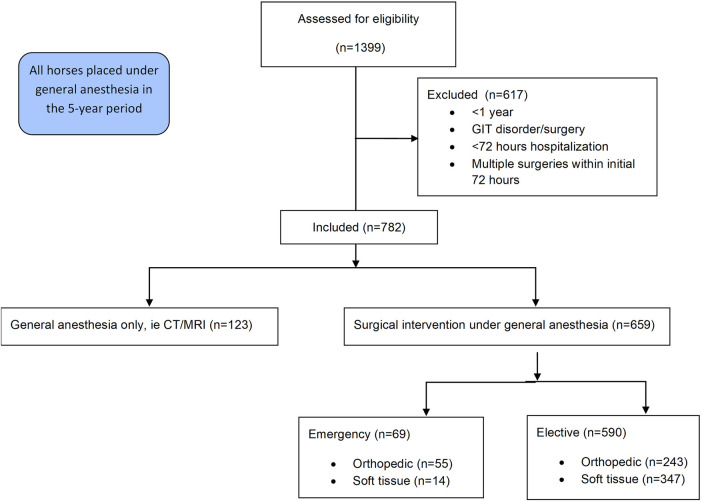
Consort flow illustrating inclusion and exclusion criteria.

This retrospective chart review includes data from all horses over the age of one year that underwent general anaesthesia at the University of Veterinary Medicine Vienna's Equine Hospital in the 5-year period from October 2013 to December 2018 and remained hospitalized at the clinic for a minimum of 72 h post-anaesthesia. Patients undergoing abdominal surgery, presenting with gastrointestinal disorders, or requiring multiple surgeries such as repeated arthroscopic lavages within the initial 72-h window, were excluded from the study (Figure 1).

The cases were stratified into an elective and an emergency group. Patients in the emergency category were not fasted due to the immediate need for surgical intervention. In contrast, horses in the elective group were fasted for a minimum of 6 h before anaesthesia.

Furthermore, both elective and emergency surgeries were categorized based on the type of procedure, distinguishing between orthopaedic and soft tissue surgeries. Horses subjected to general anaesthesia for computer tomography or magnetic resonance imaging, without concurrent surgical intervention, constituted the control group.

In addition to the horses’ age, sex, breed and weight, details of intra- and post-anaesthetic opioid regimen, including drug type, dosage, route and duration of administration, duration of anaesthesia and intraoperative blood pressure and all physical exam parameters in the 72 h following the anaesthesia were extracted from the records. Opioids were categorized into intra- and post-anaesthetic administration, with further subdivision into short-term (<24 h) and long-term (≥24 h) treatment.

Patients were classified as experiencing post-anaesthetic colic if they displayed clinical symptoms such as depression, pawing, reduced appetite, or rolling, necessitating a comprehensive colic assessment in the 72 h following anaesthesia.

### 2.2 Statistics

Data were summarized using standard descriptive statistics including mean, standard deviation, and range for continuous variables and for categorical variables frequency and proportion. Associations between categorical variables were examined by the Chi-Square test. The effect of age, sex, procedure type (anaesthesia only or surgery), emergency vs. elective surgery, anaesthesia duration, intra- and postoperative opioid administration and opioid type on PAC rate was assessed using multiple logistic regression with PAC as the outcome variable, anaesthesia without surgery as the reference level for procedure type, elective surgery as the reference level for surgery and gelding as the reference level for sex. All computations were carried out in Graphpad Prism (Version 10.0.2).

## 3 Results

A total of 782 cases, 246 (31.5%) mares, 313 (40%) geldings and 223 (28.5%) stallions met the inclusion criteria. Horses' age ranged from 1 to 30 years (mean: 9.1 years, s.d.: 6 years).

Of the 782 cases, 659 (84.3%) horses were surgical patients, the remaining 123 (15.7%) were anesthetized for diagnostic imaging purposes without concurrent surgical intervention (Table 1). The 659 surgical patients were divided into 69 (10.5%) emergency and 590 (89.5%) elective cases. The 69 emergencies included 55 (79.7%) orthopaedic and 14 (20.3%) soft tissue emergencies. In contrast, the elective cases comprised 243 (41.2%) orthopaedic and 347 (58.8%) were soft tissue surgeries. Anaesthesia duration (overall mean: 107 min, s.d.: 65 min) was significantly different between horses undergoing anaesthesia for diagnostic imaging (mean: 42 min, s.d.: 37 min), elective (mean: 115 min, s.d.: 61 min) or emergency (mean: 145 min, s.d.: 67 min) surgery (*p* < 0.0001).

**Table 1:** Number of horses receiving opioids and developing PAC by overall, intraoperative and postoperative short-term (<24 h) and long-term (>24 h) opioid administration. As horses received multiple opioids as well as intra- and postoperative opioids, cumulative percentages can exceed 100%.

		Overall		Anesthesia only		Elective surgery		Emergency surgery	
		*n* (%)	PAC *n* (%)	*n* (%)	PAC *n* (%)	*n* (%)	PAC *n* (%)	*n* (%)	PAC *n* (%)
Overall horses		782	118 (15.1)	123 (15.7)	8 (6.5)	590 (89.4)	106 (18)	69 (10.5)	4 (5.8)
Overall opioids	No opioid	34 (4.3)	3 (8.8)	2 (1.6)	0	30 (5.1)	3 (10)	2 (2.9)	0
	Opioids total	748 (95.7)	115 (15.4)	121 (98.4)	8 (6.6)	560 (94.9)	103 (18.4)	67 (97.1)	4 (6)
	Butorphanol	624	93	118	8	455	81	51	4
	Morphine	292	55	9	2	234	49	49	4
	Methadone	44	8	5	1	32	6	7	1
Intraop. opioids	No opioid	42 (5.4)	5 (11.9)	4 (3.3)	0	35 (5.9)	5 (14.3)	3 (4.3)	0
	Opioids total	740 (94.6)	113 (15.3)	119 (96.7)	8 (6.7)	555 (94.1)	101 (18.2)	66 (95.7)	4 (6.1)
	Butorphanol	589	85	116	8	437	76	36	1
	Morphine	265	46	4	0	216	42	45	4
	Methadone	4	0	0	0	2	0	2	0
Opioids <24 h	No opioid	680 (87)	103 (15.1)	110 (89.4)	6 (5.5)	527 (89.3)	94 (17.8)	43 (62.3)	3 (7)
	Opioids total	102 (13)	15 (14.7)	13 (10.6)	2 (15.4)	63 (10.7)	12 (19)	26 (37.7)	1 (3.8)
	Butorphanol	91	11	11	1	56	9	24	1
	Morphine	14	3	2	1	8	2	4	0
	Methadone	2	1	0	0	2	1	0	0
Opioids >24 h	No opioid	680 (87)	95 (14)	98 (79.7)	6 (6.1)	525 (89)	87 (16.6)	57 (82.6)	2 (3.5)
	Opioids total	102 (13)	23 (22.5)	25 (20.3)	2 (8)	65 (11)	19 (29.2)	12 (17.4)	2 (16.7)
	Butorphanol	19	1 (5.3)	19	1 (5.3)	0	0	0	0
	Morphine	47	16 (34)	3	1 (33.3)	37	14 (37.8)	7	1 (14.3)
	Methadone	38	7 (18.4)	5	1 (20)	28	5 (17.9)	5	1 (20)

The overall incidence of post-anaesthetic colic (PAC) was 15.1% (118/782, Table 1). Age, sex and anaesthesia duration had no statistically significant effect on PAC rates (Table 2).

**Table 2:** Odds ratio estimates and 95% confidence intervals (CI, *Z* and *p*-values calculated using multiple logistic regression with PAC as the outcome variable.

Variable	Odds ratio estimate	Odds ratio 95% CI	*Z*	*p*-value
Sex: mare vs. gelding	0.9334	0.5634–1.557	0.2667	0.7897
Sex: stallion vs. gelding	0.6985	0.4102–1.185	1.329	0.1839
Age	0.9822	0.9478–1.019	0.9728	0.3306
Diagnostic imaging vs. Surgery	0.3248	0.1192–0.7461	2.443	0.0146
Orthopaedic vs. soft tissue surgery	1.032	0.6265–1.703	0.1248	0.9007
Emergency versus elective surgery	4.007	1.536–13.80	2.539	0.0111
Anaesthesia duration	0.9995	0.9958–1.003	0.2443	0.8070
IntraOP opioids overall	0.6695	0.2211–1.646	0.7990	0.4243
Short-term postOP opioids overall	0.4128	0.009769–7.663	0.5616	0.5744
Short-term postOP butorphanol	2.036	0.1141–85.27	0.4559	0.6485
Short-term postOP morphine	1.322	0.1424–42.94	0.2083	0.8350
Short-term postOP methadone	0.4180	0.009406–20.28	0.4883	0.6253
Long-term postOP butorphanol	1.240	0.1896–24.46	0.1914	0.8482
Long-term postOP morphine	0.3263	0.1543–0.7086	2.897	0.0038
Long-term postOP methadone	0.7798	0.3123–2.247	0.5014	0.6161

However, horses anesthetized for diagnostic imaging purposes without concurrent surgical intervention had a significantly lower PAC rate of 6.5% (*n* = 8/123) compared to horses undergoing surgery with a PAC rate of 16.7% (*n* = 110/659, *p* = 0.0146, Table 2). Furthermore, horses anesthetized for emergency surgery had a significantly lower PAC rate of 5.8% (*n* = 4/69), than horses undergoing elective procedures with a PAC rate of 18% (*n* = 106/590) (*p* = 0.0113; Table 2). The type of surgery (soft tissue vs. orthopaedic) did not have a significant effect on PAC rate with 15.1% (45/298) orthopaedic and 18% (65/361) soft tissue surgery patients suffering from PAC (*p* = 0.9).

Overall, 34 horses (4.35%) received no opioids during their hospital stay. Of the 748 horses (95.65%) that were administered opioids, 196 (25.1%) received opioids intra- and postoperatively, 544 (69.6%) only intraoperatively and 8 (1%) only postoperatively, accumulating to 740 horses (94.6%9 with intraoperative and 204 (26.1%) with postoperative opioid treatment. Postoperative opioid therapy lasted for <24 h in 119 (58.3%) patients and long-term (≥24 h) in 85 (41.7%; Table 1).

Butorphanol dosage ranged from 0.01 mg/kg to 0.03 mg/kg intravenously as single (i.e., sedation) or repeated intravenous bolus injections. Morphine was administered at 0.1 mg/kg intramuscularly every 4–6 h. Methadone was utilized at 0.1 mg/kg intramuscularly every 4–6 h as a bolus (*n* = 43) or as part of a continuous rate infusion (CRI, *n* = 1) at a dosage of 0.017 mg/kg/h, combined with lidocaine 2% (1.2 mg/kg/h) and ketamine 10% (0.3 mg/kg/h) for post-anaesthetic pain management. No horse received methadone during anaesthesia (Table 1).

**Figure 2:**
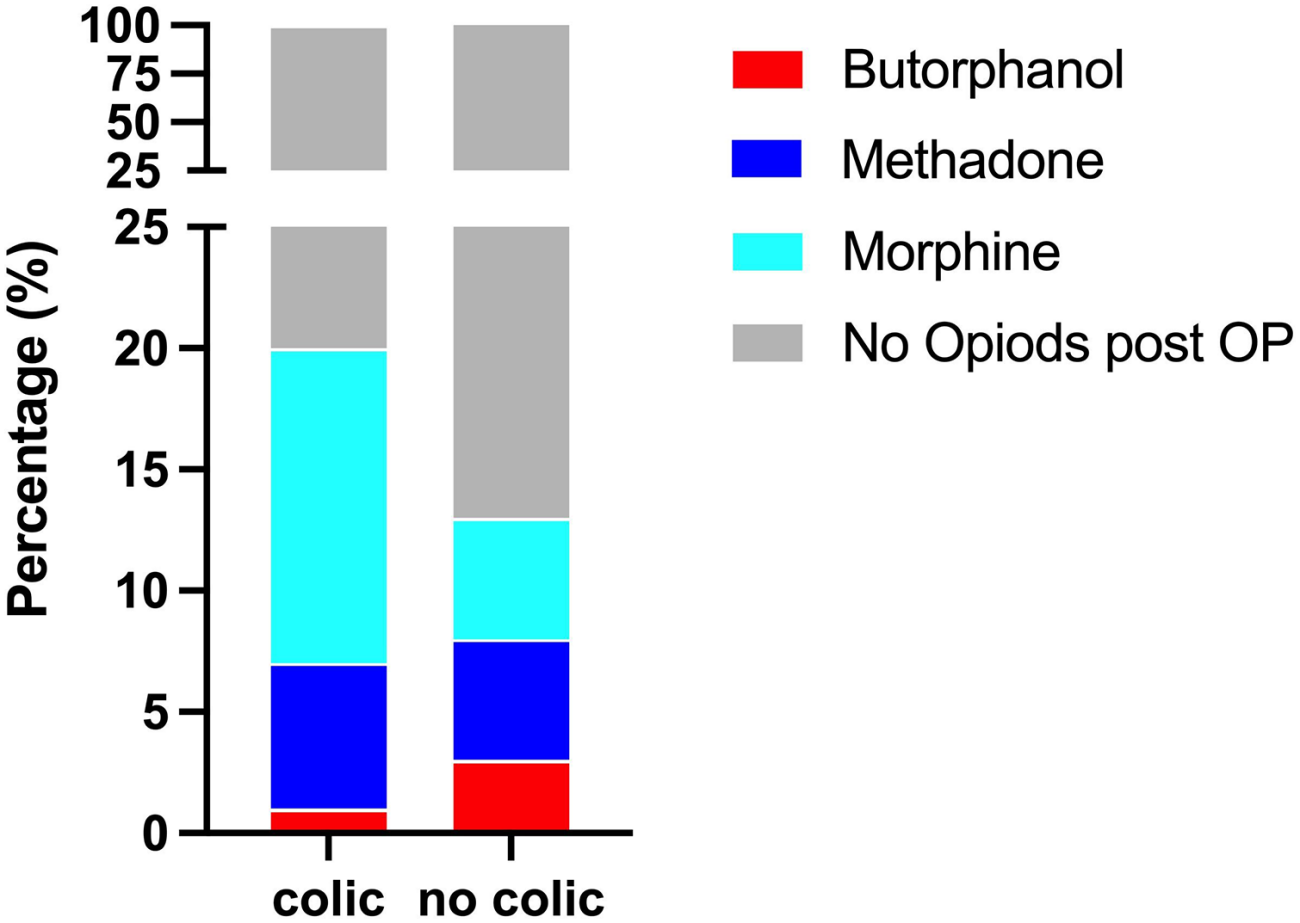
Percentage of horses receiving long-term (≥24 h) post-anaesthetic opioids with and without colic symptoms.

Neither opioids administered during anaesthesia (*p* = 0.4243) nor short-term opioid administration after anaesthesia (*p* = 0.5744) increased the incidence of PAC (Tables 1, 2). Notably, only the long-term administration of morphine resulted in a statistically significant increase of PAC (*p* = 0.0038) with 34% (16/47) horses that received morphine for ≥24 h developing PAC (Tables 1, 2, Figure 2). In contrast, long-term butorphanol [5.3% (1/19) PAC, *p* = 0.8482] and methadone [18.4% (7/38) PAC, *p* = 0.6161] had no significant effect on PAC rate, compared to horses receiving no long-term postoperative opioids [14% (95/680) PAC; Tables 1, 2, Figure 2].

## 4 Discussion

Morphine administration for longer than 24 h postoperatively emerged as the sole opioid significantly associated with increased PAC incidence in this study, in contrast to both butorphanol and methadone. This finding aligns with numerous human studies consistently ranking morphine highest in the opioid potency profile order for inducing constipation (30, 43–45). Notably, the dosage necessary for morphine's analgesic effect significantly surpasses that needed for its gastrointestinal side effects, approximately fifty-fold in humans and fourfold in experimental animals (46, 47). In horses, morphine administered intravenously at dosages as low as 0.05 mg/kg, half the dosage typically used clinically to provide analgesia, decreased propulsive gastrointestinal motility for up to 6 h and significantly reduced defecation frequency, faecal matter weight and moisture content (9, 39, 48–50).

Opioids affect gastrointestinal motility by activating the *μ*-, *κ*- and *δ*-opioid receptors of the myenteric and submucosal plexus neurons in the enteric nervous system, interstitial cells of Cajal and immune cells (13–15, 22, 51–58). Broadly, opioids induce delayed gastric emptying and constipation by disrupting neurotransmission between enteric neurons and their targets, namely, smooth muscles and epithelial cells (15). The inhibition of excitatory neurons in the myenteric plexus decreases propulsive peristalsis while the concomitant suppression of inhibitory neuromuscular transmission increases intestinal muscle tones and non-propulsive tonic contractions, which may cause abdominal cramps (13–15, 22, 51–58). Additionally, opioid-induced inhibition of submucosal secretomotor neurons diminishes epithelial secretion of Cl^−^ and osmotic water movement, exacerbating constipation (13–15, 22, 51–58).

While all three classical opioid receptor types are found in the gastrointestinal tract and contribute to analgesic and adverse effects, mechanistic studies using µ-selective drugs and µ-receptor-knockout mice indicate that opioid-induced gastrointestinal tract dysfunction is primarily mediated by the µ-receptor (13–15, 22, 51–58). Strong *μ*-agonists, such as morphine and methadone are thus more likely to induce adverse gastrointestinal side effects. Correspondingly, butorphanol, a mixed agonist-antagonist opioid analgesic with an affinity ratio for the *μ*-, *δ*-, and *κ*-opioid receptor of 1:4:25 and a a 3-, 10-, and 30-fold lower half maximal inhibitory concentration (IC50) for these receptors than morphine (38, 59), had the lowest PAC rate in this study. The lower PAC incidence following methadone compared to morphine administration observed in this study, is consistent with reported constipation relief in 80% of human patients after switch from morphine to methadone (19, 31, 60, 61). The extraopioid analgesic effects caused by methadone's non-competitive antagonist activity at the *N*-methyl-D-aspartate receptor and its function as a serotonin re-uptake inhibitor, may contribute to the lower rate of gastrointestinal effects observed with methadone compared to morphine therapy (19, 31, 60, 61). Serotonin exerts a variety of effects on intrinsic enteric neurons, extrinsic afferents, enterocytes and smooth muscle cells and its agonists are used as promotility agents to promote gastric emptying and to alleviate constipation (62–65).

Interestingly, although anaesthesia duration had no influence on PAC rate, horses anesthetized for diagnostic imaging purposes had a significantly lower PAC rate (6.5%) than horses undergoing surgery (16.7% PAC) and horses receiving no opioids postoperatively had a higher PAC (14%) rate than horses receiving butorphanol (5.3%), underscoring the importance of postoperative pain management. Pain initiates a spinal reflex arc, increases sympathetic activity and cortisol and endogenous opioid release, thus inhibiting propulsive gastrointestinal motility (66–69). Effective pain management is thus crucial to ensure patients' welfare and minimize postoperative morbidity and complications.

Notably, emergency surgeries were associated with a significantly lower PAC rate than elective surgeries in our study, which is in contrast to previous studies in which out-of-hours surgeries and horses that were not fasted before anaesthesia carried an increased risk of PAC (5, 26). The exclusion of horses with gastrointestinal problems, the more intensive supportive care and monitoring provided to emergency cases may contribute to the lower PAC rate in emergency surgeries in our study. However, horses undergoing emergency surgery, in contrast to elective procedures, also were not fasted prior to anaesthesia. Therefore, the lower PAC rate may also be attributable to the ongoing provision of food rather than the horse being presented as an emergency. Although this interpretation is more plausible from a clinical perspective, the simultaneous occurrence of these two factors does not allow statistical analysis to test this hypothesis.

The relatively high overall PAC rate in this study (15.1%), which is at the high end of the reported range of 2.8–21.1% (5, 6, 25–28), may be due to the inclusion criteria necessitating hospitalisation for 72 h post anaesthesia, which may bias toward a patient population with more severe problems and the stringent definition of colic symptoms combined with the close monitoring in our clinic.

The study's limitations are inherent in its retrospective design, the non-randomized allocation of patients to the different opioid treatment groups, the absence of both standardized pain assessment and stratification based on pain severity, and the relatively low number of patients in the diverse treatment subgroups. Hence, the selection of opioid type, administration route, and duration was determined by the anticipated or subjectively perceived level of pain, potentially leading to both over- and undertreatment of pain. Moreover, given the absence of comprehensive data regarding equipotent dosages of these opioids in equines, the employed drug dosages likely lack equipotency, thereby hindering direct comparison of their analgesic properties. Studies assessing the equianalgesic potencies of different opioids in horses and associated side effects are required. Additionally, while all three classical opioid receptor types are found in the mammalian gastrointestinal tract, their distribution patterns exhibit inter-species variability (9, 11–17, 21–23, 43, 58). For the horse only the opioid receptor distribution pattern in the small intestine has been studied (70). Given the pivotal role of colonic dysfunction in opioid-induced gastrointestinal complications across species, further investigations to determine the opioid receptor distribution in the equine colon are needed.

## 5 Conclusions

Intraoperative opioids and postoperative pain management with butorphanol and morphine did not increase the incidence of PAC in our study. Long-term (>24 h) morphine was the only opioid increasing PAC rate. In addition, patients undergoing surgery had a significantly higher PAC incidence than horses anaesthetized for diagnostic imaging and horses receiving no postoperative opioids had a higher PAC rate than those receiving butorphanol, underscoring the role of pain and pain management in PAC.

## Data Availability

The original contributions presented in the study are included in the article/Supplementary Material, further inquiries can be directed to the corresponding author.
